# Supplementary material for: What gets a dentist hired? Factors influencing employment of recent dentist graduates in Saudi Arabia
Source: PLoS One. 2025 Oct 6;20(10):e0333428. doi: 10.1371/journal.pone.0333428 (PMC12500147; doi:10.1371/journal.pone.0333428)
Supplement: S1 File — (PDF) [file pone.0333428.s001.pdf]

SN	Gender	Age (please	Nationality	Region of S	Marital stat	Type of univ	When did y	What is you
14	2	24	1	1	2	1	2024	1
15	1	25	1	4	2	1	2024	2
16	1	25	1	3	2	1	2024	2
17	1	25	1	2	2	1	2024	1
18	1	26	1	3	2	1	2023	2
19	1	27	1	1	2	1	2023	1
20	2	26	1	1	2	1	2023	1
21	1	26	1	1	2	1	2023	1
22	1	26	1	1	2	1	2024	1
23	1	25	1	1	2	1	2024	2
24	1	27	1	1	2	1	2023	1
25	1	27	1	1	2	1	2024	2
26	2	25	1	1	2	1	2024	1
27	1	25	1	1	1	1	2024	1
28	2	26	1	5	2	1	2022	2
29	1	25	1	1	2	1	2023	2
30	2	27	1	1	1	2	2021	1
31	2	26	1	1	2	1	2023	1
32	2	24	1	1	2	1	2024	1
33	1	25	1	1	2	1	2024	2
34	1	26	1	1	2	1	2022	1
35	1	27	1	1	2	1	2023	2
36	1	26	1	1	2	1	2023	2
37	2	25	1	1	1	1	2024	1
38	2	25	1	1	2	1	2024	1
39	1	25	1	1	2	1	2023	1
40	1	28	1	1	1	1	2020	2
41	2	29	1	1	2	1	2020	1
42	1	28	1	1	2	1	2022	2
43	1	26	1	1	2	1	2022	2
44	1	27	1	1	1	1	2023	2
45	1	27	1	1	2	1	2023	2
46	2	29	1	1	2	1	2020	1
47	1	25	1	1	1	1	2024	1
48	2	27	1	1	2	1	2021	1
49	2	29	1	3	2	1	2023	3
50	2	25	1	1	2	1	2023	1
51	2	26	1	1	1	1	2023	1
52	1	25	1	1	2	1	2024	1
53	2	26	1	1	2	1	2023	1
54	1	26	1	1	2	1	2024	1
55	1	28	1	1	2	1	2023	3
56	1	25	1	1	2	1	2024	2
57	2	27	1	1	2	1	2023	1
58	2	26	1	1	1	1	2023	1
59	2	29	1	1	2	1	2020	1
60	2	26	1	1	2	1	2022	2

61	2	25	1	2	2	1	2024	1
62	2	25	1	2	2	1	2024	1
63	2	30	1	3	2	2	2020	1
64	2	31	1	2	2	2	2020	3
65	1	28	1	1	2	1	2020	2
66	2	25	1	2	2	1	2024	1
67	2	25	1	2	2	1	2021	1
68	1	25	1	1	1	1	2024	2
69	1	24	1	1	2	1	2024	1
70	2	25	1	1	2	1	2024	1
71	1	24	1	1	2	1	2024	2
72	2	26	1	1	2	1	2024	1
73	2	24	1	1	2	1	2024	2
74	2	25	1	2	2	1	2023	1
75	1	27	1	1	1	1	2023	2
76	1	25	1	1	2	1	2024	2
77	1	25	1	1	2	1	2024	1
78	2	25	1	1	2	1	2024	1
79	1	26	1	2	2	1	2023	1
80	2	26	1	1	2	2	2023	2
81	2	25	1	2	1	1	2023	1
82	2	26	1	1	2	1	2024	2
83	1	30	1	1	2	1	2023	3
84	1	26	1	1	1	1	2024	1
85	2	25	1	1	2	1	2024	1
86	2	25	1	3	2	1	2024	1
87	2	29	1	1	2	2	2021	3
88	1	25	1	1	2	1	2024	2
89	1	30	1	1	1	1	2021	2
90	1	27	1	1	2	1	2021	2
91	1	30	1	1	2	1	2022	3
92	1	28	1	1	1	1	2022	1
93	1	28	1	1	1	1	2021	2
94	1	29	1	1	2	1	2021	2
95	1	28	1	1	2	1	2021	3
96	1	29	1	4	2	1	2021	2
97	1	27	1	1	2	1	2022	2
98	1	27	1	1	2	1	2024	2
99	1	28	1	1	2	1	2021	2
100	1	27	1	1	2	1	2021	3
101	1	28	1	1	2	1	2021	1
102	1	26	1	3	2	1	2023	2
103	1	27	1	1	2	1	2021	2
104	2	24	1	1	2	1	2024	2
105	2	25	1	1	2	1	2024	2
106	2	25	1	3	2	1	2023	1
107	2	27	1	3	2	2	2023	2
108	2	26	1	3	2	1	2023	1

109	2	26	1	3	2	1	2024	2
110	1	26	1	3	2	1	2022	2
111	2	26	1	3	2	1	2023	2
112	2	26	1	3	1	1	2023	1
113	1	26	1	2	2	1	2023	1
114	1	25	1	2	2	1	2023	1
115	2	27	2	3	2	2	2022	3
116	1	26	1	3	1	1	2023	2
117	2	27	1	3	2	2	2023	3
118	2	27	2	3	2	2	2022	2
119	2	25	1	2	2	1	2024	1
120	1	25	1	2	2	1	2024	1
121	1	27	1	3	1	1	2022	3
122	1	26	1	2	2	1	2024	2
123	2	25	1	2	2	1	2024	1
124	2	25	1	2	2	1	2024	1
125	2	25	1	2	2	1	2023	2
126	2	25	1	2	2	1	2024	1
127	1	33	1	3	1	2	2020	4
128	1	25	1	2	2	1	2024	1
129	1	31	1	4	1	1	2020	2
130	1	30	1	1	1	1	2020	2
131	1	33	1	1	1	1	2020	3
132	2	29	1	1	2	1	2020	2
133	2	26	1	1	2	2	2023	1
134	1	30	1	2	2	1	2020	3
135	1	29	1	3	1	2	2022	3
136	2	27	1	2	1	1	2023	1
137	2	26	1	2	2	1	2023	1
138	2	33	1	2	2	2	2020	2
139	2	25	1	1	2	1	2024	2
140	2	25	1	2	2	1	2024	2
141	1	31	1	1	1	2	2020	2
142	1	28	1	4	2	1	2022	2
143	1	27	1	1	2	2	2021	2
144	1	28	1	1	1	2	2021	2
145	1	24	1	1	2	1	2024	2
146	2	27	1	1	2	1	2022	1
147	2	29	1	2	2	2	2021	2
148	2	26	1	3	2	1	2023	1
149	2	28	1	2	1	2	2021	2
150	1	34	1	2	1	2	2020	4
151	1	25	1	2	2	1	2023	1
152	2	26	1	2	2	1	2023	1
153	2	26	1	1	2	1	2021	2
154	2	26	1	3	1	2	2021	2
155	2	24	1	1	2	1	2024	1
156	2	27	1	1	2	1	2022	2

157	1	32	1	2	2	1	2021	2
158	1	28	1	2	2	2	2023	1
160	1	30	1	2	1	2	2020	3
161	1	28	1	1	2	1	2021	1
162	2	30	1	2	2	2	2020	3
163	2	29	1	1	2	1	2021	2
164	1	25	1	1	2	1	2023	1
165	2	29	1	2	1	1	2020	1
166	2	25	1	2	2	1	2024	1
167	2	24	1	2	2	1	2024	1
168	2	26	1	2	2	1	2023	1
169	2	24	1	2	2	1	2024	1
170	1	26	1	2	2	1	2024	1
171	1	33	1	1	1	2	2020	3
172	2	31	1	2	2	2	2020	2
173	2	27	1	2	2	2	2023	2
174	2	30	1	2	2	2	2020	1
175	1	29	1	2	2	1	2020	2
176	1	27	1	5	2	1	2021	2
177	1	26	1	1	2	2	2023	2
178	2	27	1	1	1	1	2021	1
179	2	28	1	1	1	1	2021	2
180	2	29	1	2	2	2	2021	1
181	2	29	1	3	2	1	2021	2
182	1	29	1	1	2	1	2021	2
183	1	28	1	1	2	1	2020	3
184	1	28	1	1	1	2	2021	1
185	1	29	1	1	2	1	2021	2
186	2	27	1	1	2	1	2023	1
187	1	26	1	2	2	1	2023	1
188	1	27	1	3	2	1	2023	3
189	2	27	1	1	2	1	2022	1
190	2	30	1	1	2	2	2020	3
191	1	28	1	3	2	1	2020	3
192	2	27	1	5	2	1	2022	1
193	2	29	1	2	2	1	2021	2
194	1	28	1	5	1	1	2021	1
195	2	30	1	4	2	1	2020	1
196	1	30	1	1	1	1	2020	2
197	2	29	1	2	2	1	2020	1
198	2	27	1	5	2	1	2021	2
199	2	26	1	2	2	2	2023	1
200	2	29	1	4	2	1	2020	2
201	2	29	1	2	2	1	2020	1
202	2	27	1	4	2	1	2023	1
203	2	27	1	5	1	1	2023	2
204	1	27	1	4	2	1	2022	1
205	2	27	1	1	2	1	2023	2

207	2	29	1	2	2	2	2021	2
208	2	25	1	2	2	1	2024	1
209	2	26	1	3	2	1	2022	1
210	1	30	1	1	2	1	2021	2
211	2	26	1	4	2	2	2023	1
212	2	28	1	4	2	1	2022	2
213	1	26	1	1	2	1	2023	2
214	2	23	1	4	2	1	2021	1
215	1	29	1	2	2	2	2020	3
216	2	25	1	1	2	1	2022	1
217	1	26	1	2	2	1	2023	2
218	2	26	1	2	1	1	2023	1
219	1	25	1	5	2	1	2023	1
220	1	26	1	5	2	1	2023	1
221	1	26	1	2	2	1	2023	1
222	1	26	1	5	2	1	2023	1
223	2	29	1	2	2	1	2020	1
224	2	30	1	2	2	1	2021	2
225	2	27	1	1	2	1	2023	2
226	2	26	1	1	2	1	2023	2
227	2	29	1	4	2	2	2021	2
228	2	26	1	1	2	1	2023	1
229	2	26	1	1	2	1	2023	2
230	2	29	2	3	2	1	2020	2
231	1	28	1	1	2	1	2021	2
232	2	25	1	4	2	1	2023	2
234	2	29	1	2	2	2	2021	3
235	2	27	1	4	2	1	2022	2
236	1	27	1	2	2	2	2023	3
237	2	28	1	2	2	1	2021	1
238	2	26	1	4	2	1	2023	2
239	2	27	1	5	2	2	2023	1
240	2	28	1	3	2	1	2021	2
241	2	29	1	1	2	1	2020	1
242	2	25	1	1	2	1	2023	1
243	2	26	1	4	2	1	2022	1
244	1	27	1	1	2	1	2023	2
245	1	26	1	4	2	1	2023	2
246	2	27	1	2	2	1	2023	1
247	1	30	1	2	2	1	2020	1
248	2	26	1	4	2	1	2023	1
249	2	28	2	3	2	2	2021	3
250	2	27	1	4	2	1	2021	1
251	2	28	1	4	1	1	2022	1
252	2	25	1	2	2	1	2023	2
253	2	29	1	2	2	1	2020	1
254	2	27	1	2	1	1	2022	2
255	2	27	1	4	2	1	2024	1

256	2	27	1	4	2	1	2022	2
257	2	28	1	4	2	1	2024	3

Do you have	Are you par	Do you have	How many s	How much	How many t	How many t	How many t	How many t
2	1	2	0	25	1	8	1	0
1	1	2	0	10	0	0	3	0
2	1	2	1	1	1	0	1	0
2	2	2	2	4	0	0	2	0
2	2	2	2	48	3	50	2	0
2	2	2	2	5	6	6	2	0
1	2	2	1	5	6	1	2	0
2	2	2	3	5	6	20	2	0
2	2	2	4	15	2	30	1	2
2	2	2	1	1	0	35	1	0
2	2	2	2	5	2	50	1	0
1	2	2	1	3	0	7	2	4
2	2	2	1	5	0	1	1	2
1	1	2	1	20	0	2	1	2
2	2	2	2	20	1	6	3	0
2	2	2	0	10	1	10	0	2
2	2	2	2	3	0	1	2	0
2	2	1	2	10	5	30	2	0
2	2	2	0	20	0	1	1	0
2	2	2	1	15	0	0	0	0
2	2	2	6	10	7	30	2	1
2	1	2	1	4	6	50	2	3
2	1	2	2	1	5	2	2	2
2	1	2	0	6	0	0	0	0
2	2	2	0	15	0	0	1	6
1	1	2	2	50	0	0	1	50
2	1	2	1	3	1	2	2	7
2	2	2	1	10	1	0	5	0
2	1	2	1	6	19	12	0	0
2	2	2	1	30	12	20	2	0
2	1	2	0	2	1	10	0	12
2	2	2	1	5	0	0	1	0
2	2	2	1	7	2	4	3	0
2	2	2	3	10	3	50	1	0
2	2	2	2	12	1	8	2	0
2	1	2	0	3	0	27	0	0
2	2	1	4	4	0	10	2	0
2	1	2	2	2	0	3	1	0
2	2	2	2	13	1	50	1	2
2	2	2	1	15	6	50	2	5
2	1	2	2	29	0	0	2	0
1	1	2	3	4	2	10	0	2
2	2	2	1	3	1	0	0	0
1	2	2	2	7	1	20	2	0
2	2	2	3	10	6	10	2	0
1	2	2	6	10	2	0	3	0
1	1	2	2	6	3	5	2	1

2	2	2	1	30	0	0	1	0
2	2	1	0	6	3	6	1	0
2	2	2	4	5	5	0	2	0
2	2	1	0	10	10	20	0	3
2	2	2	1	5	10	5	5	3
2	2	2	0	6	0	0	0	0
2	2	1	2	10	4	50	3	3
2	1	2	1	1	2	9	0	0
1	2	2	0	3	3	1	1	0
2	2	2	0	10	0	0	0	0
2	1	2	0	20	5	30	0	0
2	2	2	0	10	1	7	1	0
2	2	1	1	3	3	7	0	0
2	2	2	2	25	5	45	2	2
2	1	2	1	2	1	10	0	3
1	2	2	0	4	0	0	0	0
2	2	2	1	4	2	1	1	0
2	2	2	0	10	1	8	1	0
2	2	2	3	5	5	1	2	0
2	2	2	0	5	1	25	2	0
2	2	2	4	12	7	8	2	0
2	1	2	1	50	0	0	0	0
1	2	2	0	5	0	0	0	1
1	1	2	0	20	3	15	0	0
2	1	2	3	9	3	0	1	0
2	2	2	1	5	5	10	0	0
2	2	2	2	50	15	30	0	3
2	2	1	0	4	2	2	0	0
2	1	2	1	2	2	2	4	0
2	2	2	1	20	9	5	3	1
1	2	2	2	3	4	10	0	1
2	1	2	2	15	12	40	3	5
2	1	2	2	3	7	5	3	0
1	2	2	1	4	0	20	0	15
1	1	2	1	2	1	3	1	0
2	1	2	2	4	1	3	2	0
2	1	2	0	9	5	20	1	0
2	1	2	0	0	0	1	0	4
1	2	2	1	18	7	1	3	0
2	1	2	0	6	5	50	2	3
2	2	2	1	6	5	5	4	0
2	2	2	2	15	5	20	2	0
1	2	2	1	12	3	3	3	0
2	2	2	0	19	1	20	0	0
2	2	1	1	12	0	0	0	0
2	2	2	0	7	3	30	2	0
2	2	2	0	0	2	33	2	0
2	2	2	1	50	3	3	1	0

2	1	1	2	50	2	11	1	2
2	2	2	2	40	1	10	2	0
1	2	2	4	25	2	20	2	0
2	1	1	1	7	1	12	2	0
1	2	2	5	7	0	8	1	0
2	2	2	2	4	0	0	1	0
2	2	2	1	2	0	0	0	0
2	2	2	2	4	15	30	5	0
2	2	2	2	7	2	30	1	8
1	2	2	2	20	0	50	0	0
2	2	2	1	3	1	1	1	0
2	2	2	3	8	0	0	1	0
2	1	1	1	19	1	20	0	0
2	1	2	0	4	0	5	1	0
1	2	1	1	6	2	40	1	0
2	2	1	1	10	0	0	1	0
2	1	2	0	10	0	20	1	0
2	2	2	0	15	1	1	1	0
2	1	2	3	0	2	4	0	0
2	2	2	2	2	2	20	1	0
1	1	2	0	5	2	1	0	0
1	1	2	4	1	3	1	2	0
1	1	2	1	0	3	10	0	1
2	2	1	3	6	6	4	5	0
2	2	2	2	3	6	35	1	0
2	2	2	1	3	34	120	0	4
2	2	2	1	2	3	6	0	1
2	2	2	2	10	0	20	2	0
2	2	2	3	25	1	120	2	20
2	2	2	2	0	20	15	2	5
2	2	2	1	9	0	0	1	0
2	2	2	1	10	0	0	1	0
2	1	2	1	1	1	40	0	2
1	1	2	3	3	2	15	2	3
2	2	1	1	3	3	5	1	0
2	2	2	2	6	0	1	0	0
2	2	2	0	10	0	30	0	0
2	2	2	3	8	3	1	2	0
2	2	2	1	5			2	0
2	2	2	3	30	2	5	2	0
2	1	1	2	5	3	50	0	0
2	2	2	1	7	2	2	0	4
2	1	2	2	4	2	10	2	0
2	2	2	2	4	0	0	1	0
2	2	2	1	6	0	2	1	0
2	2	2	2	2	2	20	1	0
2	2	2	1	11	2	16	1	0
2	2	2	1	2	2	9	3	1

2	1	2	2	10	25	78	4	0
2	1	1	1	3	5	60	2	10
2	1	2	1	1	10	100	1	0
2	2	2	2	1	5	80	2	1
2	2	1	1	4	3	100	0	10
2	1	2	0	5	9	30	1	7
1	1	2	0	3	0	0	0	4
2	2	2	2	4	0	10	1	0
2	2	2	1	6	1	30	1	0
2	2	2	3	15	1	7	1	0
2	2	2	2	20	1	30	2	0
2	2	2	2	10	5	100	1	0
2	2	2	2	20	0	0	1	0
2	2	2	5	20	10	50	5	20
1	1	2	3	10	8	8	3	5
2	2	2	6	4	1	10	1	0
2	2	2	3	5	31	20	5	3
2	2	2	1	3			3	0
2	2	2	3	2	15	80	4	0
2	1	2	2	7	8	25	2	10
2	2	2	2	10	10	20	4	4
2	2	1	1	8	4	25	3	0
1	2	1	2	20	10	35	2	1
2	1	2	1	5	1	3	1	4
2	1	2	1	2	5	15	3	0
2	1	2	2	3	5	30	2	10
2	1	1	1	0	1	1	1	1
2	2	2	1	0	3	15	0	5
1	1	2	2	4	5	10	2	0
2	2	2	1	4	1	15	1	1
2	2	2	0	1	5	50	0	2
2	2	2	1	6	3	1	2	0
2	1	2	5	1	4	15	5	0
2	2	2	1	4	20	5	0	0
2	2	1	1	6	1	1	2	0
2	2	2	1	3	3	10	4	0
2	1	2	3	3	4	2	3	0
2	1	2	3	7	4	3	5	0
2	1	2	2	6	23	32	4	5
2	2	2	3	5	1	1	2	0
2	1	2	2	7	3	8	3	7
2	1	2	2	20	8	21	1	5
2	2	2	1	3	3	20	3	10
2	2	2	2	8	1	2	3	0
2	2	2	2	3	4	12	2	0
2	2	2	1	4	3	1	2	0
2	2	2	3	5	0	0	1	0
2	2	1	2	6	0	0	1	0

2	2	2	2	20	5	3	2	1
1	2	2	4	30	0	30	1	0
2	2	2	2	50	2	15	2	0
2	1	2	1	2	2	7	1	2
2	2	2	3	10	1	1	1	0
2	2	2	1	2	1	10	0	0
2	2	2	1	10	7	15	2	0
2	2	2	1	2	0	0	0	0
2	2	2	2	5	3	160	0	15
2	2	1	2	15	3	35	3	0
2	2	2	5	49	10	90	2	10
2	2	1	3	20	3	100	2	0
2	2	2	2	7	6	2	2	0
2	2	2	2	2	5	1	1	2
1	2	2	1	3	9	50	2	0
2	2	2	2	3	3	9	0	0
2	2	2	1	6	3	1	1	0
2	2	2	1	3	5	3	1	0
2	1	2	1	1	0	10	1	0
2	2	2	1	2	2	35	2	0
2	1	2	1	20	2	2	4	0
1	2	2	1	12	1	5	2	0
2	2	2	1	4	0	10	0	0
2	2	2	2	6	0	20	5	1
2	1	2	2	10	1	1	3	0
2	2	2	2	2	1	10	2	0
2	1	2	1	1	3	9	1	0
2	1	2	3	4	0	1	2	0
2	2	1	2	14	0	5	1	0
2	1	2	3	5	0	0	1	0
2	2	1	2	2	0	1	0	0
2	2	1	1	3	4	6	2	0
2	2	2	2	2	2	10	2	0
2	2	2	2	3	1	0	3	0
2	2	2	2	20	4	60	2	10
2	2	2	1	1	3	5	0	0
1	2	2	1	11	0	1	0	2
2	1	2	5	4	0	1	0	0
2	1	2	4	13	1	11	2	0
2	1	2	6	12	0	1	1	0
1	2	2	3	8	6	3	1	2
2	2	1	2	5	0	1	0	0
2	1	2	1	5	1	0	1	0
2	2	1	3	5	1	20	3	0
2	2	2	1	23	3	1	1	0
2	2	2	6	10	2	5	1	1
1	1	2	1	0	0	0	0	0
2	2	1	4	2	10	10	1	2

2	2	2	3	2	1	5	4	0
2	2	2	3	6	4	35	2	0

Please ans	Please ans	Please ans	Please ans	Please ans	Please ans	Please ans	Please ans	Please ans
1	1	2	1	2	1	2	2	2
2	2	2	2	2	2	2	2	1
1	1	1	1	1	2	2	2	2
1	1	2	1	2	2	2	2	2
1	1	2	1	2	1	2	2	2
1	1	1	1	1	1	1	1	1
1	1	2	1	2	2	2	2	2
1	1	2	1	2	2	2	2	2
1	2	2	1	2	2	2	2	2
1	1	2	1	2	2	2	2	2
1	1	2	2	2	2	2	2	2
1	1	1	1	2	2	2	2	2
1	1	1	1	1	1	1	2	2
1	1	2	1	2	1	1	2	2
1	1	2	1	2	2	2	2	2
1	2	1	1	2	2	2	2	2
2	2	2	1	2	2	2	2	2
1	1	2	1	2	2	1	2	2
1	2	1	1	2	2	2	2	2
1	1	2	1	2	2	2	2	2
1	1	2	1	2	2	2	2	2
1	1	2	1	2	2	2	2	2
1	1	2	1	1	2	1	2	2
2	2	2	2	2	2	2	2	2
1	2	2	1	2	2	2	2	2
1	1	1	1	1	1	1	1	1
2	2	2	2	2	2	1	2	2
1	1	2	2	2	2	2	2	2
1	1	2	1	2	2	2	2	2
1	1	1	1	2	2	2	2	2
2	1	2	1	2	2	2	2	1
1	1	2	1	2	2	2	2	2
1	1	2	2	2	2	2	2	2
2	1	1	1	1	1	1	1	1
1	2	2	1	2	2	2	2	2
2	2	1	1	2	2	1	2	2
1	1	1	1	2	2	2	2	2
2	2	2	2	1	2	2	2	2
1	1	2	1	2	2	2	2	2
1	1	2	1	2	2	2	2	2
2	2	2	2	1	1	1	1	1
1	1	1	1	2	1	1	2	2
1	1	2	2	2	1	2	2	2
1	1	2	2	2	2	2	2	2
1	1	2	1	2	2	2	2	2
2	2	2	2	2	1	2	2	2
1	1	2	1	2	2	2	2	2

1	1	1	1	2	2	2	2	2
2	2	2	1	2	2	2	2	2
1	1	2	1	2	1	2	2	2
2	2	2	2	2	2	2	2	2
1	1	2	1	2	2	2	2	2
1	1	2	1	2	1	2	2	2
1	1	2	1	2	2	2	2	2
1	1	2	1	1	2	2	2	2
1	1	2	1	2	2	1	2	2
1	2	2	1	1	2	2	2	2
1	1	2	1	1	2	1	2	2
1	2	1	1	2	2	2	2	2
1	2	1	2	2	2	2	2	2
1	2	2	1	2	2	2	1	2
1	1	2	1	2	1	2	2	2
1	1	2	1	1	1	2	2	2
1	1	2	2	2	1	2	2	2
1	1	2	1	2	1	2	2	1
1	1	2	1	2	2	2	2	2
1	1	2	2	2	2	2	2	2
1	1	2	1	2	2	2	2	2
2	2	1	1	1	2	1	2	2
2	2	2	2	1	1	1	1	2
1	1	2	1	2	2	2	2	2
1	1	2	1	2	2	2	2	1
1	1	1	1	1	1	1	1	1
1	1	2	1	2	2	2	2	2
1	1	2	1	2	1	2	1	1
1	1	2	1	2	2	2	2	2
1	1	1	1	2	2	2	2	2
1	1	1	1	1	2	1	2	2
1	1	1	1	1	1	2	2	2
2	2	2	1	2	2	2	2	2
1	1	2	1	2	2	1	2	2
1	2	2	1	2	2	2	2	2
1	1	1	1	2	2	2	1	2
1	1	2	1	2	2	2	2	2
1	1	2	1	2	1	2	2	2
1	1	1	1	2	2	2	2	2
1	1	1	1	2	2	2	2	2
1	1	1	1	2	2	2	2	2
1	1	2	1	2	1	2	2	2
1	1	2	2	2	2	2	2	2
1	2	2	1	1	1	1	2	2
1	1	1	1	2	2	2	2	2
1	1	2	1	2	2	1	2	2
1	1	2	1	2	2	2	2	2
1	1	1	1	2	2	2	2	2

1	2	2	2	2	2	2	2	1
1	1	1	1	2	2	2	2	2
2	2	2	2	2	2	2	2	2
2	1	1	1	1	1	2	2	2
1	2	2	1	2	1	2	2	2
1	1	2	1	2	2	2	2	2
2	2	2	2	2	2	1	1	2
1	1	2	1	2	1	2	2	2
1	2	2	1	2	2	2	2	2
1	2	1	1	1	2	1	2	2
2	2	1	1	2	2	2	2	2
1	1	1	1	2	1	2	2	2
1	2	2	1	2	1	2	2	2
1	1	2	2	1	2	2	2	2
1	2	1	1	2	1	2	2	2
1	1	2	1	1	2	2	2	2
2	2	1	1	2	2	2	2	2
2	2	1	1	1	2	2	2	2
1	1	1	1	2	2	2	2	2
2	2	2	1	2	2	2	2	2
1	1	2	1	2	2	2	2	2
1	1	2	1	2	1	2	2	2
1	1	2	1	2	2	2	2	2
1	2	1	1	1	1	1	1	1
1	2	2	1	1	1	1	2	2
1	1	2	1	2	2	2	2	1
1	1	2	2	2	2	2	2	2
2	2	2	2	2	2	2	2	2
1	2	2	1	2	2	1	2	2
1	1	2	1	1	2	2	2	2
2	2	2	1	2	1	1	2	2
2	2	1	1	1	2	2	2	2
1	1	2	1	2	2	2	2	2
1	1	2	1	2	2	2	2	2
2	1	2	1	1	2	2	2	2
1	1	2	1	2	2	2	2	2
2	1	2	1	2	1	2	2	2
1	2	2	1	2	2	2	2	2
1	1	2	1	2	2	2	2	2
1	2	2	1	2	2	2	2	2
1	2	2	1	2	2	2	1	2
1	1	2	1	2	1	2	2	2
1	1	2	1	2	1	2	2	2
1	1	2	1	2	2	2	2	2
1	1	2	2	2	2	2	1	2
1	1	1	1	2	1	2	1	2
1	1	2	1	2	2	2	2	2
1	2	2	1	2	2	2	2	2

1	1	2	1	2	2	2	2	2
2	2	2	1	2	2	2	2	2
1	1	2	1	2	2	2	2	2
1	1	1	1	2	2	2	2	2
2	2	2	1	2	2	2	2	2
1	1	2	1	2	2	1	2	2
1	1	2	1	2	2	1	2	2
2	2	2	1	2	2	2	2	2
2	2	2	1	2	2	1	2	2
1	1	2	1	2	1	1	2	2
1	1	2	2	2	2	2	2	2
1	1	2	1	2	2	2	2	2
1	1	1	1	2	2	2	2	2
1	2	2	1	2	2	2	2	2
1	1	2	1	2	2	2	2	2
2	2	2	2	2	2	2	2	2
1	1	2	1	2	2	2	2	2
1	1	2	1	2	2	2	2	2
1	1	2	2	2	1	2	2	2
1	1	1	1	2	2	2	2	1
1	1	2	1	2	2	2	2	2
1	1	2	2	2	1	1	2	2
1	2	2	1	2	2	2	2	2
1	1	1	1	2	1	1	2	1
1	1	2	1	2	1	2	2	2
1	2	2	1	2	2	2	2	2
2	2	2	2	2	2	2	2	2
1	1	2	2	2	2	2	2	2
2	2	2	1	2	2	2	2	2
1	1	1	1	1	1	2	2	2
1	1	1	1	2	2	2	2	2
2	2	2	1	2	2	2	2	2
1	1	2	1	2	2	2	2	2
1	1	2	1	2	2	2	2	2
1	1	2	2	2	2	2	2	2
1	2	2	1	2	2	2	2	2
1	1	2	1	2	2	2	2	2
1	1	2	2	2	2	2	2	2
2	2	2	1	2	2	2	2	2
1	2	2	2	2	2	2	2	2
2	2	2	2	2	2	2	2	2
2	1	2	1	2	1	2	2	2
1	1	2	2	2	2	2	2	2
1	1	2	1	2	1	2	2	2
1	1	1	1	2	1	1	2	2
1	1	2	1	2	2	2	2	2
1	1	2	1	1	1	2	2	2
1	2	2	1	2	2	2	2	2

1	1	2	1	2	2	2	2	2
2	2	1	1	1	2	2	2	2
2	2	1	1	2	2	2	2	2
1	2	2	1	2	2	2	2	2
2	1	2	1	2	2	2	2	2
2	2	2	2	2	2	2	2	2
1	1	1	1	2	2	2	2	2
2	1	1	1	2	2	1	2	2
1	1	2	1	2	2	2	2	2
1	1	2	1	2	2	2	2	2
1	1	2	1	2	2	2	2	2
1	1	2	2	2	1	2	2	2
1	2	1	1	2	2	2	2	2
1	2	2	2	2	2	2	2	2
1	2	1	1	1	2	1	2	2
2	2	2	1	1	2	2	2	2
2	2	2	2	2	2	2	2	2
1	2	2	1	2	2	2	2	2
2	2	2	1	2	1	1	2	2
1	1	1	1	2	2	2	2	2
1	2	2	1	2	2	2	1	2
2	2	2	1	2	2	1	2	2
2	1	2	1	2	2	2	2	2
1	1	2	2	2	2	2	2	2
1	1	1	1	1	1	1	1	1
1	2	2	1	2	2	2	2	2
2	2	1	1	2	2	1	2	2
1	1	2	2	2	2	2	2	2
1	1	2	1	2	2	2	2	2
2	2	2	1	2	2	2	2	2
1	2	2	1	2	2	2	2	2
1	1	2	1	2	2	2	2	2
1	2	2	1	2	1	2	2	2
1	2	2	2	2	1	2	2	2
1	1	2	1	2	2	1	2	2
1	2	2	1	2	2	2	1	2
1	1	1	1	1	1	1	2	2
2	2	2	1	2	2	2	2	2
1	1	2	2	2	1	2	2	2
1	1	2	1	2	2	2	2	2
2	2	2	1	2	2	2	2	2
1	2	2	1	2	2	2	2	2
1	2	2	2	2	2	2	2	2
1	2	2	2	2	2	2	2	2
2	2	1	1	1	1	1	1	1
1	2	2	1	2	2	2	2	2
2	1	2	2	2	1	1	1	1
1	1	1	1	2	2	2	1	2

2	2	2	1	2	2	2	2	2
1	1	1	1	2	2	1	2	2

Where do y	Where do y	Where do y	Where do y	Where do y	Where do y	What is the	What is a fa	Do you con
1	1	1	1	2	2	7000	6000	2
1	2	1	2	2	2	7500	2000	1
1	1	1	2	2	2	15000	15000	2
1	1	1	1	2	2	6000	8000	5
1	2	1	2	2	2	7000	10000	1
1	1	1	1	1	1	18000	18000	2
1	1	1	2	1	2	7000	10000	1
1	2	1	2	2	2	7000	7000	2
1	1	2	2	1	2	7000	15000	1
2	2	2	1	2	2	15000	9000	1
1	1	1	2	2	2	7000	9000	2
1	1	2	2	2	2	7000	12000	1
1	1	1	1	2	2	5000	8000	1
1	1	1	1	1	2	7000	18000	5
1	1	2	2	1	2	7000	7000	1
1	1	1	2	2	2	7000	9000	1
1	1	1	1	2	2	20000	10000	1
1	1	1	2	2	2	6000	8800	1
1	2	1	2	2	2	7000	7000	2
1	2	1	2	1	2	10000	9000	2
1	2	1	1	2	2	12000	12000	1
1	2	1	1	2	2	12000	15000	5
1	2	1	2	2	2	8000	7000	5
1	2	2	2	2	2	10000	10000	2
1	1	1	2	2	2	9000	10000	1
1	1	1	1	1	1	9000	12000	1
1	2	2	1	2	1	3000	12000	2
1	1	1	2	2	2	15000	15000	2
1	2	1	1	1	2	7000	9000	1
1	2	1	2	1	2	7500	12000	2
1	2	2	2	2	2	7000	13000	5
1	1	2	2	2	2	7000	8000	2
1	1	1	1	1	2	7000	10000	2
1	1	1	1	1	2	3000	7000	1
1	2	1	2	2	2	7000	9000	1
1	1	2	2	2	2	7000	7000	1
1	2	1	2	2	2	7000	7000	2
1	1	1	1	2	2	7000	9000	2
1	1	1	1	2	2	7500	7000	1
1	2	1	2	1	2	7000	13000	5
1	1	2	1	2	2	19000	12000	1
1	1	2	1	2	2	5000	8000	1
1	1	2	2	1	2	9000	10500	2
1	2	1	2	2	2	7000	5000	2
1	2	1	2	2	2	8000	10000	2
1	2	1	2	2	2	15000	18000	1
1	1	1	2	2	2	15000	13000	1

1	2	1	1	2	2	10000	13000	5
1	1	1	1	1	2	7000	12000	1
1	2	1	2	1	2	10000	12000	2
1	1	2	1	2	2	12000	15000	2
1	2	1	2	2	2	10000	10000	1
1	1	1	1	2	2	6000	8000	1
1	2	1	2	2	1	7000	7000	5
1	1	1	2	2	2	7000	9000	2
1	1	2	2	1	2	9190	13000	1
1	1	1	2	2	2	7000	9000	2
1	1	1	1	1	2	7000	7000	1
1	1	1	2	2	2	7000	10000	1
1	1	2	2	1	2	7000	5000	2
1	1	2	2	1	2	10000	90000	1
1	1	2	2	1	2	7000	12000	1
1	1	1	1	2	2	10000	10000	5
1	1	1	1	1	2	7000	9000	2
1	1	1	1	1	2	9000	10000	1
1	2	1	2	2	2	10000	12000	2
1	2	1	2	2	2	8000	8000	1
1	1	1	1	2	2	9000	10000	2
1	1	1	1	2	2	4000	4000	1
1	2	2	2	2	2	7000	10000	2
1	1	1	1	1	2	9000	12000	2
1	1	1	1	2	2	5000	7000	1
1	1	2	1	2	2	9000	7000	2
1	2	1	1	1	2	10000	10000	1
1	1	1	2	2	2	8000	8000	2
1	1	1	2	2	2	12000	8000	2
1	1	1	2	1	2	7000	12000	1
1	1	1	1	2	2	7000	5000	2
1	1	1	1	1	2	5000	12000	2
1	1	1	1	2	2	10000	5000	2
1	1	2	1	1	2	7500	7000	2
1	1	2	2	2	2	15000	8000	1
1	2	2	2	2	2	5000	10000	2
1	2	1	2	2	2	9000	7000	1
1	1	1	1	2	2	7000	13000	1
1	2	1	1	2	2	10000	10000	1
1	1	1	1	2	2	9000	10000	1
1	1	1	1	2	2	7000	5000	2
1	2	1	2	2	2	7000	10000	1
1	1	2	1	1	2	9000	7000	1
1	1	1	1	1	2	5000	8000	1
1	1	1	2	2	2	7000	9000	1
1	1	1	2	2	2	7000	9200	1
1	1	1	1	1	1	7000	9000	1
1	2	1	2	2	2	7000	18000	1

1	2	1	2	2	2	7000	9000	1
1	1	1	1	2	2	9000	12000	1
1	2	1	2	2	1	7000	12000	2
2	1	2	2	2	2	8000		2
1	1	1	2	2	2	8000	7000	5
1	1	2	2	1	2	20000	20000	2
1	1	2	1	1	2	4000	3000	1
1	1	1	1	1	2	7000	15000	2
2	1	1	1	2	2	6000	7000	1
1	1	1	2	2	2	7000	7000	1
1	2	1	1	1	2	10000	10000	1
1	1	2	2	2	2	15000	15000	1
1	1	2	2	2	2	7000	9000	1
1	1	1	1	2	2	7000	7000	1
1	1	1	1	2	2	5000	7000	2
1	1	1	2	2	2	10000	13000	2
1	1	2	2	2	2	7000	70000	2
1	1	1	1	2	2	10000	10000	2
1	1	1	2	2	2	7000	10000	2
1	1	1	2	2	2	7000	9000	1
1	1	1	1	1	1	10000	18000	1
1	2	1	1	2	2	8000	12000	1
1	1	1	1	2	2	15000	12000	2
1	2	2	1	2	2	15000	14000	1
1	2	1	1	1	2	6000	8000	2
1	1	2	1	1	2	7000	13000	1
1	2	1	2	1	2	10000	12000	2
1	2	1	2	1	2	8000	8000	2
1	2	1	2	1	2	7000	7000	1
1	2	1	2	1	2	10000	8000	5
1	1	2	1	1	2	5000	4000	1
1	1	1	2	1	2	7000	7000	1
1	2	1	2	2	2	6300	10000	2
1	2	1	1	1	2	8000	2000	1
1	2	1	2	1	1	18000	18000	1
1	1	1	2	2	2	10000	7000	1
1	1	2	2	2	2	5000	9000	1
1	1	1	1	2	2	7000	7000	1
1	2	2	1	2	2	7000	7000	1
1	1	1	2	2	2	7000	10000	1
1	2	1	2	2	2	9000	9000	1
1	2	1	1	1	2	13000	12000	2
1	1	2	1	2	2	7000	7000	1
1	1	1	2	2	2	30000	15000	1
1	1	1	1	2	2	7000	6000	1
1	1	2	1	2	2	7000	12000	1
1	1	1	2	2	2	7000	7000	1
1	1	1	1	1	2	5000	7000	2

1	2	1	2	2	2	6317.5	15000	1
1	2	1	2	2	2	9200	9200	2
1	2	1	2	2	2	10000	10000	5
1	2	1	2	2	2	4000	12000	2
1	1	1	1	1	2	7000	15000	2
1	2	2	1	1	2	7000	8000	2
1	1	1	1	1	2	7000	10000	1
1	1	2	2	2	2	25000	20000	1
1	1	1	1	1	2	5000	6000	1
1	1	2	1	1	2	8000	8000	2
1	2	1	1	2	2	7000	10000	1
1	1	1	1	2	2	7000	12000	2
1	2	2	2	1	2	9000	16000	2
1	2	2	1	2	2	18000	25000	2
1	1	1	1	1	2	20000	15000	1
2	2	2	2	2	2	7000	5000	1
1	2	1	2	2	2	7000	18000	1
1	2	1	1	1	2	7000	7000	5
1	1	1	1	2	2	10000	9000	2
1	2	1	2	1	2	7000	7000	1
1	2	1	1	1	2	10000	8000	2
1	2	1	2	2	2	5000	12000	1
1	1	1	1	1	2	8000	15000	5
1	1	1	1	1	2	7000	10000	5
1	1	1	1	2	2	7000	7000	2
1	2	1	1	1	2	12000	7000	2
2	2	2	2	2	2	1000	1000	5
1	2	1	2	2	2	8000	8000	2
1	2	1	2	2	2	7000	9000	1
1	1	1	1	2	2	7000	14000	1
2	1	2	2	2	2	3500	9000	1
1	1	1	1	2	2	7000	10000	1
1	1	1	1	2	2	7000	5000	1
1	1	1	2	2	2	12000	12000	1
1	1	2	2	2	2	7000	10000	1
1	1	2	1	2	2	10000	12000	1
1	1	1	2	2	2	12000	14000	1
1	2	1	2	1	2	30000	16500	2
1	1	1	1	1	2	9000	11000	1
1	2	1	2	2	2	3500	20000	1
1	2	1	1	1	2	8000	5000	1
1	1	1	1	2	2	8000	12000	1
1	2	1	1	1	2	10000	10000	2
1	1	1	2	2	2	10000	10000	1
1	1	1	1	1	2	6000	9000	1
1	1	1	1	2	2	15000	12000	1
1	1	1	1	2	2	10000	10000	1
1	1	1	2	2	2	15000	15000	1

1	1	1	1	2	2	8000	5000	1
1	2	2	2	2	2	8000	12000	1
1	1	1	2	2	2	7000	9000	1
1	1	2	2	1	2	9000	9000	1
1	2	1	2	2	2	7000	7000	1
1	1	2	2	2	2	7000	7000	1
1	1	1	1	2	2	7000	15000	2
1	2	1	1	2	2	7000	12000	1
1	2	1	2	1	2	15000	12000	1
1	1	1	2	2	2	7000	7000	1
2	2	2	1	2	2	7000	100000	1
1	1	1	1	2	2	8000	7000	1
1	2	2	2	2	2	8000	8000	2
1	2	1	2	1	2	7000	10000	2
1	1	1	1	2	2	7000	9000	2
1	1	1	2	2	2	6000	7000	1
1	1	1	1	2	2	15000	20000	2
1	1	1	1	1	2	7000	10000	2
1	1	1	2	2	2	7000	7000	1
1	1	1	2	2	2	6000	7000	2
1	1	1	1	1	1	8000	8000	1
2	1	1	2	1	2	7500	7500	1
1	2	1	1	2	2	8000	8000	2
1	1	1	1	2	2	7000	7000	1
1	2	1	1	2	2	7000	12000	2
1	1	1	1	1	2	7000	7000	1
1	2	2	2	2	2	7000	10000	1
1	1	1	1	2	2	7000	10000	1
1	1	1	2	2	2	7000	12000	1
1	1	2	1	2	2	15000	20000	1
1	2	1	2	2	2	7000	4000	1
1	1	1	1	2	2	7000	10000	1
1	2	1	2	2	2	12000	7000	5
1	1	1	1	2	2	7000	10000	1
1	1	1	1	2	2	7000	8000	2
1	2	1	2	1	1	10000	9000	1
1	1	1	2	1	2	7000	3000	1
1	1	1	2	2	2	8000	15000	1
1	1	1	2	2	2	7000	9000	1
2	1	2	2	2	2	20000	12000	1
1	1	2	2	2	2	7000	9000	1
1	1	2	1	2	2	6000	10000	1
1	2	1	2	2	2	10000	9000	1
1	2	1	2	1	2	7000	7000	2
1	1	1	2	2	2	7000	5500	1
2	1	2	2	1	2	20000	12000	1
1	2	1	2	1	1	8000	8000	2
1	1	1	1	2	1	10000	10000	1

1	1	1	1	1	2	8000	12000	1
1	1	1	1	2	2	2000	10000	1

Do you con	Do you con	Do you con	Do you con	How many t	How many t	How many t	How many t	How many t
2	2	5	2	1	1	1	5	1
1	1	1	5	1	2	1	1	5
2	2	2	2	1	1	1	1	1
1	1	1	5	5	1	1	1	5
1	2	2	2	1	1	1	1	1
2	2	2	2	1	1	1	1	1
1	1	2	2	1	1	1	1	1
2	2	2	2	1	1	1	1	1
2	2	2	2	1	1	1	1	1
1	2	1	2	2	2	1	1	2
2	2	2	5	1	1	1	1	5
1	2	5	5	1	1	1	5	5
1	2	1	5	1	1	1	1	5
5	5	2	5	5	5	5	3	5
1	1	1	5	1	1	1	1	5
2	2	2	2	1	1	1	1	1
1	2	2	1	2	2	1	1	2
2	2	5	2	1	1	1	5	1
2	2	5	5	1	1	1	5	5
2	2	5	5	1	1	1	5	5
1	2	2	5	1	1	1	1	5
2	2	2	5	5	1	1	1	5
5	5	5	5	5	5	5	5	5
2	2	5	2	1	1	1	5	1
1	5	1	5	1	1	5	1	5
1	1	1	5	2	2	2	1	5
5	1	5	1	2	5	2	5	2
2	2	2	2	1	1	1	1	1
2	5	5	5	1	1	5	5	5
2	1	2	2	1	1	1	1	1
5	5	5	2	5	5	5	5	2
2	2	5	5	1	1	1	5	5
2	2	5	2	1	1	1	5	1
5	2	5	5	1	5	1	5	5
5	2	5	5	2	5	1	5	5
5	2	1	2	1	5	1	1	1
2	1	5	2	1	1	1	5	1
2	1	5	1	1	1	1	5	1
2	2	1	5	1	1	1	1	5
5	5	5	5	5	5	5	5	5
1	2	2	2	1	1	1	1	2
2	2	5	5	1	1	1	5	5
1	2	5	5	1	1	1	5	5
2	2	5	2	1	1	1	5	1
2	2	2	2	1	2	1	1	1
2	1	2	2	1	1	1	1	1
1	1	1	5	1	2	1	1	5

5	5	5	5	5	5	5	5	5
1	2	1	1	1	1	1	1	2
2	2	2	2	1	1	1	1	1
2	2	1	5	1	1	1	1	5
2	2	2	5	1	1	1	1	5
1	1	5	5	1	1	1	5	5
5	5	5	5	5	5	5	5	5
2	1	1	1	1	1	1	1	1
2	1	2	2	1	1	1	1	1
2	2	2	2	1	1	1	1	1
2	2	1	2	1	1	1	1	1
5	1	1	5	1	5	1	1	5
2	2	2	2	1	1	1	1	1
1	5	1	5	1	1	5	1	5
2	1	2	2	1	1	1	1	1
5	5	1	5	5	5	5	1	5
1	2	2	5	1	1	1	1	5
5	5	1	5	1	5	5	1	5
2	2	2	2	1	1	1	1	1
1	5	5	5	2	2	5	5	5
2	2	1	2	1	1	1	1	1
2	2	5	5	1	1	1	5	5
2	2	2	2	1	1	1	1	1
2	2	1	5	1	1	2	1	5
1	2	2	1	1	1	1	1	1
1	1	2	2	1	1	1	1	1
2	2	1	2	1	1	1	1	1
2	2	5	2	1	1	1	5	1
2	2	2	2	1	1	1	1	1
2	5	2	5	2	1	5	1	5
2	2	1	2	1	1	1	1	1
2	2	2	2	1	1	1	1	1
1	1	2	1	1	1	1	1	1
2	1	2	2	1	1	2	1	1
5	1	5	5	1	5	2	5	5
2	2	1	5	1	1	1	1	5
2	2	2	2	1	1	1	1	1
2	1	5	1	1	1	1	5	1
1	1	1	5	1	1	1	1	5
2	2	2	2	2	1	1	1	1
1	1	2	1	1	1	1	1	1
1	2	1	5	2	2	1	1	5
2	2	2	2	1	1	1	1	1
2	1	1	1	1	2	1	1	1
1	2	5	1	1	1	1	5	1
5	5	1	5	1	5	5	1	5
1	1	2	1	1	2	1	1	1
2	2	2	2	1	1	1	1	1

1	2	2	2	1	1	1	1	1
1	1	2	1	1	1	2	1	1
2	2	2	2	1	1	1	1	1
2	2	2	2	1	1	1	1	1
5	5	5	5	5	5	5	5	5
1	1	5	2	1	1	1	5	1
5	2	5	5	2	5	1	5	5
2	2	1	2	1	1	1	1	1
1	2	1	2	1	1	1	1	1
1	1	1	1	2	2	2	2	3
2	5	2	5	1	1	5	1	5
1	2	2	2	1	1	1	1	1
2	2	2	2	2	2	1	1	1
5	2	2	5	1	5	1	1	5
2	2	2	2	1	1	1	1	1
2	2	2	2	1	1	1	1	1
5	5	2	5	1	5	5	1	5
1	1	2	1	1	1	1	1	1
2	1	2	1	1	1	3	1	1
5	2	1	2	1	5	1	1	1
1	1	1	1	2	1	1	1	1
1	2	2	5	1	1	1	1	5
5	2	2	5	1	5	1	1	5
1	1	1	1	1	1	1	1	1
1	2	1	5	1	1	1	1	5
2	1	5	5	2	1	1	5	5
1	2	1	2	1	1	1	1	1
2	2	2	2	1	1	1	1	1
5	5	1	5	1	5	5	1	5
5	5	5	5	5	5	5	5	5
2	1	2	1	2	2	1	1	1
2	2	1	1	2	1	1	1	1
2	2	2	2	1	1	1	1	1
2	2	1	2	1	1	1	1	1
1	1	5	1	2	1	1	5	2
1	1	1	5	1	1	1	1	5
2	2	1	2	1	1	1	1	1
2	2	1	5	2	1	1	1	5
1	5	2	5	1	1	5	1	5
2	2	1	5	1	1	1	1	5
2	1	2	2	3	1	1	1	1
1	1	2	1	1	3	2	1	3
2	5	1	5	1	1	5	1	5
5	5	1	5	1	5	5	1	5
5	1	1	5	1	5	1	1	5
1	1	1	2	2	1	1	1	1
2	2	2	5	1	1	1	1	5
5	5	2	5	1	5	5	1	5

1	1	1	5	1	1	1	1	5
2	1	2	1	1	1	1	1	1
5	5	1	5	5	5	5	2	5
1	2	2	2	1	1	1	1	1
2	2	2	2	1	1	1	1	1
2	2	2	2	1	1	1	1	1
1	1	1	1	1	1	1	1	1
5	5	1	5	2	5	5	2	5
1	2	2	1	1	1	1	1	1
2	2	2	2	1	1	1	1	1
1	1	1	1	1	2	1	1	1
1	2	1	2	1	2	1	1	1
2	2	2	2	1	1	1	1	1
2	2	2	2	1	1	1	1	1
1	1	1	1	1	1	1	1	1
1	1	1	1	1	1	1	1	1
1	5	1	5	3	3	5	3	5
5	2	5	5	5	5	1	5	5
2	2	1	2	1	1	1	1	1
1	5	1	5	1	1	5	1	5
2	1	2	5	1	1	1	1	5
2	1	2	2	1	1	1	1	1
5	5	5	5	5	5	5	5	5
5	1	5	5	5	5	3	5	5
2	2	2	2	1	1	1	1	1
2	2	2	2	1	1	1	1	1
5	5	5	5	5	5	5	5	5
2	5	1	5	1	1	5	1	5
2	1	1	1	1	1	1	1	1
2	2	1	2	1	1	1	1	1
2	2	5	1	1	1	1	5	1
2	1	2	1	1	1	1	1	1
5	2	1	5	1	5	1	1	5
1	1	1	5	1	1	1	1	5
1	2	5	5	1	1	1	5	5
2	5	2	5	1	1	5	1	5
1	1	5	2	1	1	1	5	1
2	2	2	2	1	1	1	1	1
2	2	1	1	2	1	1	2	3
2	2	2	2	1	1	1	1	1
1	1	5	1	2	1	2	5	2
1	5	1	1	2	2	5	1	1
2	2	2	2	1	1	1	1	1
2	5	2	5	1	1	5	1	5
2	2	2	2	1	1	1	1	1
1	5	1	5	1	2	5	1	5
2	2	2	2	1	1	1	1	1
2	1	5	1	3	1	1	5	1

2	1	1	2	2	2	1	1	1
2	2	2	2	1	1	1	1	1
1	2	1	5	1	1	1	1	5
1	2	5	5	1	1	1	5	5
5	5	5	5	1	5	5	5	5
1	1	2	1	1	1	1	1	1
2	2	5	2	1	1	1	5	1
1	1	5	5	1	1	1	5	5
2	1	1	2	2	2	1	1	2
2	2	1	2	1	1	1	1	1
1	2	2	2	1	1	1	1	1
1	2	2	1	2	1	1	1	1
1	2	2	2	1	2	1	1	1
1	1	1	1	1	1	1	1	1
2	2	2	2	1	1	1	1	1
1	5	5	5	1	1	5	5	5
2	2	2	5	1	1	1	1	5
2	2	5	5	1	1	1	5	5
5	5	5	5	1	5	5	5	5
2	2	1	2	1	1	1	1	1
2	1	1	1	1	1	1	1	1
1	2	2	2	1	1	1	1	1
5	2	5	2	1	5	1	5	1
2	2	2	5	1	1	1	1	5
2	2	5	5	1	1	1	5	5
1	5	1	5	1	1	5	1	5
1	1	5	1	1	1	1	5	1
2	2	5	5	1	1	1	5	5
1	1	1	1	1	1	1	1	1
2	2	1	2	1	1	1	1	1
1	1	1	1	2	2	2	2	2
5	5	5	5	1	5	5	5	5
5	5	1	5	5	5	5	1	5
5	5	1	5	1	5	5	1	5
2	2	1	2	1	1	1	1	1
5	1	2	2	1	5	2	1	1
1	1	1	1	1	1	1	1	1
2	1	2	2	1	1	1	1	1
1	2	1	2	1	1	1	1	1
5	5	5	1	2	5	5	5	2
2	1	1	1	1	1	1	2	2
2	1	2	1	1	1	1	1	1
1	1	5	2	1	1	1	5	1
2	2	2	2	1	1	1	1	1
1	5	1	1	1	1	5	1	1
5	5	5	5	1	5	5	5	5
2	2	1	2	1	1	1	1	1
1	1	1	1	3	2	2	2	2

2	2	2	2	1	1	1	1	1
2	1	2	1	1	1	1	1	1

Which of th	Which of th	Which of th	Which of th	Which of th	Which of th	Which of th	Which of th	Which of th
2	2	1	1	1	2	2	1	2
1	1	2	2	1	1	2	2	2
2	2	2	2	2	2	1	1	1
1	2	2	1	1	2	2	2	2
1	1	2	2	1	1	1	1	2
2	2	2	2	1	2	2	2	1
2	2	1	1	1	2	2	1	2
2	2	2	1	1	1	2	2	2
2	2	2	1	1	2	2	2	2
2	2	2	1	1	1	2	2	2
2	2	1	2	1	1	1	1	2
2	2	2	1	2	2	2	2	2
2	2	2	1	1	2	2	2	2
2	1	1	1	1	1	1	1	2
2	2	2	1	1	2	2	2	2
2	1	2	1	1	1	1	1	2
2	1	1	1	1	2	2	1	2
2	2	1	1	1	2	2	2	2
2	2	2	2	1	2	2	2	2
2	2	1	1	1	1	1	1	1
1	1	2	1	1	1	1	2	2
1	2	2	2	2	1	1	1	1
1	2	2	2	1	2	1	1	2
2	2	2	2	2	2	2	2	2
2	2	1	1	1	2	2	2	2
1	1	1	1	1	1	1	1	1
1	2	1	1	1	1	1	1	1
2	2	1	1	1	2	2	2	2
2	2	2	2	1	1	1	1	1
2	2	2	2	1	1	2	1	2
2	1	2	1	2	1	1	1	1
2	1	2	1	2	2	2	2	2
2	2	2	2	2	2	2	2	2
1	2	1	1	1	1	1	1	1
1	2	2	2	1	2	2	2	2
1	2	2	2	1	1	2	2	1
2	2	2	1	1	2	2	2	2
1	2	2	1	1	1	2	2	2
2	2	2	1	1	2	2	1	2
2	2	2	2	1	2	2	2	2
1	1	1	1	1	2	2	2	2
1	1	2	1	1	2	2	1	2
1	2	2	1	1	1	2	2	1
2	2	2	1	1	1	1	2	1
2	2	2	1	1	2	2	2	2
2	2	1	1	1	2	2	2	2
1	1	1	1	1	1	2	1	1

2	2	2	2	2	2	2	2	2
2	1	1	1	1	2	2	1	2
2	2	1	1	1	2	2	2	2
2	2	2	1	2	1	1	1	1
2	2	2	1	1	2	2	2	2
2	2	2	2	1	2	2	2	2
2	1	1	2	1	2	2	2	2
2	2	2	1	1	1	1	1	1
2	2	2	2	1	1	2	1	2
1	1	1	2	1	1	1	1	1
2	2	1	1	1	1	1	1	2
2	2	1	1	1	2	2	1	2
2	2	2	1	2	2	2	2	2
1	2	1	1	1	1	2	1	2
1	2	2	2	1	2	1	1	2
1	2	2	2	1	1	1	1	1
2	2	2	2	1	1	2	1	2
2	2	1	1	1	2	2	1	2
2	2	2	2	1	2	2	2	2
2	1	1	1	1	1	1	1	2
2	2	2	2	1	1	1	1	2
1	1	1	1	1	1	1	1	1
1	1	1	1	1	1	1	1	1
1	1	2	1	1	1	1	2	1
1	1	1	1	2	1	1	1	1
2	1	2	2	1	2	2	2	2
2	1	1	1	1	1	1	1	1
2	2	2	2	2	2	2	2	2
2	2	2	2	1	1	1	2	2
2	2	1	1	1	2	2	2	2
2	2	2	1	1	2	1	1	2
2	2	2	1	1	1	1	1	1
1	2	2	1	1	1	1	1	2
1	2	2	2	1	1	1	2	2
2	2	2	2	1	1	1	1	1
2	2	2	2	2	1	1	1	1
2	1	2	2	1	2	2	2	2
1	1	1	2	1	1	1	1	1
2	2	2	1	1	2	2	2	2
2	2	2	2	1	1	1	1	2
2	2	2	2	2	2	2	2	2
2	1	1	1	1	2	2	2	2
1	1	1	1	1	1	2	1	2
2	1	1	2	2	1	2	2	2
2	2	2	2	1	2	2	2	2
2	2	2	2	1	2	2	2	2
2	1	1	1	1	2	2	2	2
1	2	2	1	1	1	2	2	2

1	2	2	1	1	1	1	1	2
2	2	2	2	1	2	2	2	2
2	2	2	2	1	2	2	2	2
2	2	2	2	1	2	2	2	2
2	2	2	1	1	2	2	2	2
2	2	2	2	1	1	2	1	1
2	1	2	1	1	2	2	2	2
1	2	2	2	1	1	1	2	2
1	1	2	1	1	2	2	1	1
1	1	2	2	1	1	1	1	1
1	2	1	1	1	2	2	2	2
2	2	2	1	1	2	2	2	2
2	2	2	2	1	2	2	2	2
2	2	1	1	1	2	2	2	2
2	2	2	2	1	2	2	2	2
2	2	2	2	1	1	1	1	2
2	2	1	1	1	2	2	1	2
2	2	2	1	1	2	2	2	2
1	1	1	1	1	1	1	1	1
2	2	2	2	1	2	1	2	2
1	2	2	2	1	2	2	2	2
2	2	2	2	2	2	2	2	1
2	2	2	1	1	1	1	1	2
1	1	2	1	1	2	2	2	1
2	2	2	1	1	1	2	2	1
1	2	2	1	1	1	1	1	1
1	2	2	1	1	1	2	2	1
2	2	2	1	1	2	2	2	2
2	2	1	1	1	1	2	2	1
2	2	2	2	2	2	2	2	2
2	1	2	1	1	1	2	1	2
1	2	2	1	1	2	2	2	2
2	2	2	2	2	2	2	2	2
2	2	2	1	1	1	1	1	1
2	2	2	1	2	2	2	2	2
2	2	2	1	1	1	1	1	2
2	1	1	1	1	2	2	1	2
2	2	2	1	1	2	2	2	2
2	2	1	2	1	1	2	1	2
2	1	1	1	1	2	2	2	2
2	2	2	2	1	2	1	1	2
2	1	2	1	1	1	1	1	1
2	2	2	1	1	1	1	1	2
2	2	1	1	1	1	1	1	2
2	2	2	2	1	1	1	1	1
2	2	2	1	1	2	2	2	2
2	2	2	1	1	2	2	2	2
2	2	2	2	2	2	2	2	2

1	2	2	2	1	1	1	1	1
2	2	2	1	1	2	2	2	2
1	2	2	2	1	1	2	2	2
2	2	2	1	1	2	2	2	2
2	2	2	1	2	2	2	2	2
1	2	2	2	1	1	2	2	1
1	1	1	1	1	1	1	1	1
2	2	1	1	1	2	2	2	2
2	2	2	2	2	2	1	1	2
1	2	2	2	1	1	2	2	2
1	1	1	1	1	1	1	1	1
2	2	2	2	1	1	1	1	2
2	2	2	2	1	1	1	1	1
1	2	1	1	2	2	2	2	2
2	2	2	2	1	1	1	1	1
2	2	2	2	2	2	2	2	2
1	2	2	2	1	2	2	2	2
2	1	1	1	1	2	1	1	2
2	2	2	2	1	2	2	2	2
2	2	2	2	1	1	1	1	1
2	2	2	1	1	2	2	2	2
2	2	2	1	1	2	2	2	2
1	1	1	1	1	1	1	1	1
2	1	1	1	1	1	1	1	2
2	2	2	1	1	1	1	1	1
1	2	2	1	1	1	1	1	1
1	1	1	1	1	1	1	1	1
2	2	2	1	1	2	2	2	2
2	2	1	1	1	2	2	2	2
2	2	2	1	1	2	2	2	2
2	2	2	1	1	1	1	1	2
2	1	2	1	1	2	2	2	2
2	2	2	1	1	1	2	2	2
1	2	2	1	1	1	1	1	1
2	2	2	2	1	2	2	2	2
2	2	2	2	2	2	2	2	2
2	2	2	1	1	1	2	2	2
2	2	2	2	1	1	2	2	1
2	1	1	1	1	2	1	1	2
2	2	1	2	2	2	2	2	2
1	1	1	1	1	1	1	1	1
1	1	1	1	1	1	1	1	1
1	2	2	1	1	2	2	2	2
2	2	2	2	1	2	2	2	2
2	1	1	1	1	2	2	1	2
1	1	1	1	1	1	1	1	1
2	2	2	1	1	2	2	2	2
1	1	1	1	1	1	1	1	1

1	1	1	1	1	1	2	1	1
1	2	2	1	1	2	2	1	2
2	2	2	1	1	1	1	2	2
1	2	1	1	2	2	2	2	2
1	2	2	1	1	2	2	2	2
2	2	1	1	1	2	2	2	2
1	2	2	1	1	2	2	2	2
2	1	1	1	1	1	2	2	2
1	2	2	2	1	2	2	2	2
2	2	1	1	1	2	2	2	2
2	2	2	2	1	2	2	2	2
2	1	1	1	1	1	1	1	1
2	2	2	2	2	2	2	2	2
2	2	2	2	1	1	2	2	1
2	2	2	1	1	2	2	1	2
2	2	2	2	1	2	2	2	2
2	2	2	1	1	2	2	2	2
2	2	2	1	1	2	2	2	2
1	1	1	1	1	2	2	2	2
1	2	2	1	1	2	2	2	2
2	2	2	2	1	2	2	2	2
2	1	1	1	1	2	2	1	2
2	2	1	2	1	1	2	2	1
1	2	2	1	1	2	2	1	2
2	1	2	1	1	2	1	1	1
1	1	1	1	1	2	2	2	2
1	2	1	1	1	2	1	1	2
2	2	2	1	2	2	2	2	2
2	1	1	1	1	1	1	1	1
2	1	1	1	1	2	2	2	2
2	2	2	1	2	1	2	1	2
1	2	2	2	1	2	2	2	2
2	2	1	2	1	1	1	1	1
2	2	2	2	2	2	2	2	2
2	2	2	2	2	2	1	1	2
2	1	1	1	1	1	1	1	1
1	1	2	1	1	1	1	1	2
2	2	2	1	1	2	1	2	1
2	2	2	2	1	2	2	2	2
2	1	1	1	1	1	1	1	1
1	1	1	1	1	1	1	1	1
2	2	2	2	2	1	2	1	1
2	2	2	1	2	2	2	2	2
2	2	2	2	1	2	2	2	2
2	1	1	1	1	1	1	1	1
1	2	2	1	1	1	2	2	1
2	2	1	2	1	2	2	2	1
1	1	1	1	1	1	1	1	2

1	2	2	1	1	1	2	2	1
1	1	1	1	1	1	1	1	1

Please desc	What is you	Your curren	If you are w	Are you satisfied with your salary/income?
4				
4				
1	1	1	2	2
1	6	1	4	2
2	5	1	4	1
1	1	1	1	1
1	1	1	1	1
2	1	1	1	2
2	1	2	3	2
4				
1	1	2	3	2
4				
4				
2	1	2	3	1
3				
4				
1	1	1	1	1
1	1	2	3	2
4				
1	2	1	3	2
2	1	2	1	2
1	2	2	1	2
2	1	2	1	2
4				
4				
1	6	1	4	2
1	1	2	3	2
1	1	1	1	1
1	1	2	3	2
3				
1	1	1	1	1
4				
1	1	1	1	1
4				
2	1	1	1	1
1	1	2	1	1
2	1	1	1	1
4				
2	1	2	1	1
1	1	2	1	2
4				
3				
4				
1	2	2	3	2
1	1	1	1	1
2	1	1	1	1
1	1	2	1	2

4				
4				
1	1	1	1	1
1	1	2	1	2
1	1	2	1	2
4				
3				
1	1	1	3	2
4				
4				
4				
4				
4				
4				
2	1	2	1	2
4				
4				
4				
1	5	1	4	1
1	1	2	1	2
4				
4				
3				
1	2	3	3	2
4				
4				
1	1	2	3	2
4				
1	1	2	3	2
2	1	1	1	1
3				
2	1	2	3	2
1	1	2	1	2
2	2	2	1	2
2	1	2	2	1
1	1	1	1	1
2	1	2	1	2
3				
1	1	2	1	2
2	1	2	3	2
4				
2	1	1	1	1
2	1	2	3	2
1	4	2	1	2
4				
1	1	2	1	1
1	1	2	3	1
1	1	2	3	2

1	1	2	1	2
2	1	1	1	1
1	1	2	3	2
1	2	2	2	2
1	1	2	3	2
4				
4				
1	2	2	1	2
4				
1	1	2	2	1
4				
4				
1	1	2	3	2
1	1	2	1	1
4				
4				
4				
1	1	3	3	1
1	1	2	3	2
1	1	2	1	2
1	1	2	3	1
1	1	2	3	1
2	1	2	1	2
1	1	1	1	2
1	1	2	1	2
2	1	2	3	2
1	1	2	1	2
3				
3				
1	1	1	1	1
4				
1	1	1	3	1
1	1	2	3	2
3				
2	1	2	3	2
1	1	2	3	1
4				
4				
3				
1	1	2	3	2
2	1	2	3	2
1	1	2	1	2
2	1	1	3	1
1	1	1	1	1
1	1	2	1	2
1	1	2	3	2
4				
3				

2	1	2	1	2
4				
3				
1	1	2	2	2
2	1	2	2	1
2	4	2	1	2
4				
1	1	2	1	1
4				
1	2	2	3	2
2	1	3	1	1
4				
1	1	1	1	1
1	1	1	3	2
3				
2	1	2	1	1
1	1	2	3	2
2	2	2	3	2
4				
1	1	2	3	2
2	1	1	1	1
1	1	2	1	1
1	1	2	1	1
1	1	2	1	2
1	1	2	1	2
2	1	2	1	2
2	1	2	3	2
3				
3				
1	1	2	3	1
2	1	2	3	2
1	1	1	1	1
3				
2	1	2	3	2
1	1	2	3	1
2	1	2	1	2
1	1	1	1	1
1	1	1	1	1
2	1	2	3	1
1	1	1	1	1
1	1	3	3	2
3				
1	1	1	1	1
2	1	1	1	1
1	1	2	1	2
1	2	1	1	2
1	1	1	3	1
4				

1	1	2	3	1
1	1	2	1	2
1	1	2	3	2
2	1	2	1	2
1	1	2	1	2
3				
1	2	2	1	2
4				
2	1	2	1	2
1	1	2	1	2
2	1	2	3	1
1	1	1	1	1
2	1	1	1	1
4				
2	1	1	1	1
1	1	2	3	1
1	1	2	1	2
1	1	2	3	2
4				
4				
1	1	2	3	1
4				
1	4	2	1	2
2	1	2	3	2
1	1	1	1	1
4				
1	1	2	1	2
1	1	1	1	1
1	1	2	3	1
1	1	1	1	1
4				
2	1	2	2	1
3				
1	1	1	1	1
4				
1	1	2	1	2
1	1	2	3	2
1	1	1	2	1
2	1	2	1	1
2	1	2	3	1
2	1	2	3	1
3				
1	1	1	1	1
1	1	1	1	1
1	1	1	3	1
1	2	2	2	2
4				
2	1	2	1	2

2	1	2	3	2
1	2	2	2	2
