# Supplementary material for: What gets a dentist hired? Factors influencing employment of recent dentist graduates in Saudi Arabia
Source: PLoS One. 2025 Oct 6;20(10):e0333428. doi: 10.1371/journal.pone.0333428 (PMC12500147; doi:10.1371/journal.pone.0333428)
Supplement: S2 File — (PDF) [file pone.0333428.s002.pdf]

Factors affecting hiring recent dental graduates in Saudi Arabia

Protocol Title: Factors affecting hiring recent dental graduates in Saudi Arabia
Study Sponsor: Not applicable.
Chief Investigator: Dr Khalid Aboalshamat
You are invited to participate in the study because you are
1) a dentist who graduated in the last 5 years (2019 to 2023).
2) spent at least 6-months after finishing the internship year.

(Please do not participate if you did not finish your intern years).
3) lives in Saudi Arabia.
4) not retired.
Aims of the study: Assess the factor affecting hiring recent dental graduates in Saudi Arabia.
Number of expected participants: 385
Participation is voluntary.
You are completely free to choose whether to join the study or not. Your decision will not affect your relation by the research team.
If you decide to participate in this study, you will go through the following procedures: Answer the questionnaire.
Expected benefits: Helping the stakeholders and your graduates fellow to assess

the factor affecting hiring recent dental graduates in Saudi Arabia.
Expected risks: Not applicable.
Freedom of withdrawal:
You have the right of withdrawal from the study at any time without explaining the cause of withdrawal.
Study revision and approval:
This study will be revised and approved by a special board of experts at the Institutional Review Board-IRB or research ethics committee.
How information and results will be used: The result of this study will be published in a scientific journal or conference to be used for public knowledge.
Confidentiality: Your name will not be used in this study, and each participant will have a numerical code.
For further information, you can contact

Dr Khalid Aboalshamat
Consent statement

I hereby confirm that all aspects related to the study have been explained to me including aims and procedures and that my participation is voluntary with no extra-expenses and there is no payment offered to me for participation.

By Click next, you agree on the term of this consent form.

khalidaboalshamat@gmail.com Switch account

Not shared

Factors affecting hiring recent dental graduates in Saudi Arabia

khalidaboalshamat@gmail.com Switch account

Not shared

* Indicates required question

Demographic data

Please answer the following questions according to the best answer to you.

Gender *

- Male
- Female

Marital status: *

- Married
- Non-married

What is your employment status? *

- I have a job.
- I am jobless

Your current job sector: *

- Governemental
- Private
- Both
- I am not currently employed

Do you have other source of income but not reated to you as a dentist? *

- Yes
- No

How many patients you see in a day (write a number please)? *

Your answer

Are you financially responsible about family members (family, children or parent, etc...). *

- Yes
- No

Age (please write in number) *

Your answer

Where do you live (city name) *

Your answer

Region in Saudi Arabia *

- Western
- Central
- Southern
- Eastern
- Northern

Nationality *

- Saudi
- Non-Saudi

University of graduating type: *

- Private
- Governmental

What is the name of the university that you have studies dentistry at? *

Your answer

When did you finished your internship year?

- ○ 2019
- ○ 2020
- ○ 2021
- ○ 2022
- ○ 2023

What is your GPA in graduation? *

- ○ Excellent (A)
- ○ Very good (B)
- ○ Good (C)
- ○ Pass (D)

How many scientific paper did you publish? *

Choose ▾

How many volunteering work did you participated in before? *

Choose ▾

Do you consider yourself active on the following social media platforms? *

	I do not have account	Yes	No
Twitter (X)	○	○	○
Snapchat	○	○	○
LinkedIn	○	○	○
TikTok	○	○	○

How many followers do you have on the following social media platforms? *

	I do not have account	>1,000	1,000 to 5,000	>5,000
Twitter (X)	○	○	○	○
Snapchat	○	○	○	○
LinkedIn	○	○	○	○
TikTok	○	○	○	○

How many time did you apply for dentist job in the governmental sector? *

Choose ▾

How many time did you apply for dentist job in the privet sector? *

Choose

How many time did you apply postgraduate position in the Saudi Commission of Health specialties (SCHS)? *

Choose

Did you apply for other jobs rather than dentistry? *

- Yes
- No

Please answer the following questions before you got a dental job *

	Yes	No
Were you willing to move to another city for the dental job?	○	○
Were you ready to work extra hours per day WITHOUT extra salary?	○	○
Were you ready to work extra hours per day WITH extra salary?	○	○
Were you comfortable with a delay in salary for 2 months?	○	○
Were you willing to work without the needed tools?	○	○
Were you willing to work as a dental assistant?	○	○
If your duty manager asked you to do an intervention that you were not specializing in, would you do it?	○	○
If your duty manager asked you to do an intervention that you were not licensed to do (such as orthodontic treatement), would you do it?	○	○
Were you willing to work in an area that you do not like?	○	○

What is your preference for working as a dentist? *

	Yes	No
Governmental	○	○
Private	○	○
Academic	○	○
Non-dental field	○	○

Do you have a senior dentist as a relative in your family? *

- ○ Yes
- ○ No

Which of the following skills do you have?

	Yes	No
Accounting	○	○
social media inflencer	○	○
Content creator	○	○
Photography	○	○
Communication skills	○	○
Business skills	○	○
Sales skills	○	○
Marketing skills	○	○
Entrepreneurs skills	○	○

What is the minimal salary you are ready to work with as a dentist in the private sector (please write a number)?

Your answer

Back Next Clear form

Never submit passwords through Google Forms.

This content is neither created nor endorsed by Google. Report Abuse - Terms of Service - Privacy Policy

Google Forms

Factors affecting hiring recent dental graduates in Saudi Arabia

khalidaboalshamat@gmail.com Switch account

Not shared

* Indicates required question

For employed

What is your employment status (mainly)? *

- Full time dentist
- Part time dentist
- Dental clinic owner
- Full time, but not as a dentists
- Part time, but not as a dentists
- Owner of a business, but not a dental clinic
- Dental assistant

Are you satisfied with your salary?

- Yes
- No

Back Submit Clear form

Never submit passwords through Google Forms.

This content is neither created nor endorsed by Google. Report Abuse - Terms of Service - Privacy Policy

Google Forms
