# Supplementary material for: What gets a dentist hired? Factors influencing employment of recent dentist graduates in Saudi Arabia
Source: PLoS One. 2025 Oct 6;20(10):e0333428. doi: 10.1371/journal.pone.0333428 (PMC12500147; doi:10.1371/journal.pone.0333428)
Supplement: Attachment — Submitted filename: Response to Reviewers.docx [file pone.0333428.s005.docx]

Dear editor,

We would like to thank the reviewers and the editorial team for their valuable time and constructive feedback on our manuscript. We appreciate the thoughtful comments and suggestions, which have helped us improve the quality and clarity of our work. Below, we provide a point-by-point response to each comment and describe the corresponding revisions made to the manuscript. All the changes were highlighted by yellow in the revised version.

| **Comment** | **Response** |
| --- | --- |
| **Reviewer 1** |  |
| Thank you for the opportunity to review the manuscript titled “What Gets a Dentist Hired? Factors influencing employment of recent dentist graduates in Saudi Arabia”. The study addresses a relevant and timely issue, particularly in the context of rising numbers of dental graduates and employment challenges in the health sector. While the authors attempt to contribute meaningfully to the literature by identifying employment correlates among Saudi dental graduates, there are notable methodological and interpretive issues that require attention before the manuscript can be considered for publication. | Thank you. |
| Overall Assessment The study employs a cross-sectional design using a self-developed questionnaire distributed via social media to recent dental graduates. While the topic is important, several concerns limit the generalizability and interpretive power of the findings. These concerns relate primarily to sampling design, insufficient methodological clarity, and overreaching in data interpretation. | Thank you for your feedback. All the points were addressed in the following rows, with specific concentration on the issue in the manuscript. |
| Methodological Concerns and Clarity: The authors justify their use of convenience sampling due to a lack of a sampling frame, but do not adequately explain the rationale behind the four-month post-graduation inclusion criterion. | This point was explained now in the manuscript:  “The requirement of a minimum of 4 months post-graduation was applied because, at the time of recruitment, the most recent graduates at year of 2024 in all universities in Saudi Arabia, had just completed their internship year and were eligible to apply for jobs. This ensured that only fully graduated dentists were included and prevented potential confusion among individuals who had not yet officially graduated.”. |
| Further, the recruitment approach, described as distribution via social media, is vague. It remains unclear whether participants were contacted through individual outreach, open group posts, or targeted advertising. This has implications for understanding who was likely to respond and how representative the sample is of all recent graduates. | Thank you for your comment. This was now explained in detailed in the following:  “Recruitment primarily involved identifying and joining groups that included dentists in Saudi Arabia, either through keyword searches or referrals from colleagues and friends. Study invitations were then shared within these groups, both through open posts and direct messages to individual members. In addition, potential participants were identified on platforms such as Twitter/X and LinkedIn by searching relevant keywords and reviewing user profiles or connections, after which invitations were sent privately to dentists who met the inclusion criteria. No paid or sponsored advertising was used.” |
| The description of questionnaire development is similarly ambiguous. The manuscript does not clearly identify which items were adapted from existing studies and which were developed by the authors. | Thank you for your comment. This is true. The answer is here:  Demographic and employment items (age, sex, city, internship/graduation year, GPA, employment status/sector, time to first job, income, satisfaction) were adapted from Farag et al. [16]. Career-preference and family-influence items (preferred work setting; presence of a senior-dentist relative) were adapted from Halawany et al. [17]; perceived career-challenge constructs (e.g., obtaining a government post, establishing a clinic) were adapted from Fita et al. [6]; and practice-plan constructs were informed by Zhao et al. [5]. All remaining items were developed by the authors. These items included number of publications/volunteering and job applications (government, private, Saudi Commission for Health Specialties, and non-dental); willingness/constraint items (relocation, rural work, extra hours with/without pay, salary delay, limited tools, working as a dental assistant, unlicensed procedures); social-media activity/followers; soft-skills/entrepreneurship; and income model (fixed, percentage, or both).  However, we did not include this lengthy details, instead, in the new revised manuscript only these items that were only generated by the authors to make it more concise in the following:  “These items included including number of publications/volunteering and job applications (government, private, Saudi Commission for Health Specialties, and non-dental); willingness/constraint items (relocation, rural work, extra hours with/without pay, salary delay, limited tools, working as a dental assistant, unlicensed procedures); social-media activity/followers; soft-skills/entrepreneurship; and income model (fixed, percentage, or both).”   Please let us know, if you prefer to have all the details. |
| References to a group of five dental professionals involved in item generation lack context, such as their expertise or affiliations. | Thank you, for the comment, and it was clarified in the manuscript now:  “(one professor of dental public health and four dental interns)” |
| Terms like “classified according to the results” also need clarification. | The sentence was rephrased again into:  “The questions were corrected and adjusted multiple times for clarity and then organized into appropriate sections of the questionnaire.” |
| Additionally, although the authors claim content validity was established through a pilot with 17 professionals, more detail on the outcome of that process is needed. | This was explained now:  “Feedback led to simplify items’ wording and instructions, expand and standardize response options to avoid confusion and overlapping. Some items were removed based on the feedback of the long original form of the questionnaire to avoid response burnout. Other items were reordered to improve logic and flow.” |
| Questionable Inferences and Over-Interpretation The manuscript makes several claims that extend beyond what the sampling and analysis can support. For example, it states that individuals with higher GPAs were “more likely” to be employed in government positions. Given the non-probabilistic sampling, such language implies causality or population-level generalizability that is not warranted. | Thank you for the comment. We adjusted all statement of “more likely” into association statement in the whole text related to these variables. |
| In other instances, findings like the association between marital status and employment are interpreted in ways that are culturally and temporally ambiguous (e.g., suggesting marriage may lead to employment or vice versa), without acknowledging reverse causation or third variables. | This is an important point. It was explained now in the discussion now:  “This association should be interpreted cautiously, as reverse causation or other cultural confounding could explain this relationship. Therefore, the results indicate correlation within this sample and cannot implies causation.” |
| Similarly, findings from bivariate tests are discussed as definitive patterns rather than preliminary associations, and comparisons to earlier studies (e.g., Farag et al.) are made without considering differences in design, sample population, or recruitment approach. | Thank you for the comment, we have add multiple sentences to highlight this point, including:  “In this convenience sample, all results reflect bivariate associations and should be read as preliminary. They are not causal and not population-level estimates.”  “It should be noted that comparisons with prior work, especially Farag et al. [16], should be interpreted cautiously because of differences in design, sampling frame, years of recruitment.”  “Furthermore, the study was based on a self-reported questionnaire, especially those related to soft skills, job applications, and social media activity. Also, as categorical variables were assessed using bivariate tests, multivariable analyses using larger sample size would be required to assess whether the associations persist after adjustment.” |
| Measurement and Validity Issues Several variables—particularly those related to soft skills, job applications, and social media activity—are based on self-report without any control for social desirability or independent validation. | This was addressed now in the discussion as a limitation:  “However, it is important to note that our assessment was self-reported, and it is not known how participants rated themselves as having sales skills or not. Further assessment methods and instruments can be used to measure these variables objectively in future research.”  And  “Furthermore, the study was based on a self-reported questionnaire, especially those related to soft skills, job applications, and social media activity. Also, as categorical variables were assessed using bivariate tests, multivariable analyses using larger sample size would be required to assess whether the associations persist after adjustment.” |
| This is especially important given the nature of questions such as “Would you do a procedure you’re not licensed to perform?”.  The ethical implications of such items are concerning, and the framing of these questions warrants reconsideration. | This was clarified now in the methodology by:  “Some sensitive questions were added based on real field events, despite thier ethical sensitivity, such as “If your clinical manager asked you to do a procedure you were not licensed to do (e.g., orthodontic treatment), would you do it?”. This ethically sensitive item was reframed to measure perceived pressure rather than willingness, with anonymous responses interpreted as exploratory.” |
| The authors should also reflect on how reliable these self-assessments are and how they affect conclusions drawn about employability. | This was addressed in multiple responses above, and here is examples of that:  “In this convenience sample, all results reflect bivariate associations and should be read as preliminary. They are not causal and not population-level estimates.”  “It should be noted that comparisons with prior work, especially Farag et al. [16], should be interpreted cautiously because of differences in design, sampling frame, years of recruitment.”  “Furthermore, the study was based on a self-reported questionnaire, especially those related to soft skills, job applications, and social media activity. Also, as categorical variables were assessed using bivariate tests, multivariable analyses using larger sample size would be required to assess whether the associations persist after adjustment.” |
| Contradictory or Misleading Interpretations The manuscript includes internal contradictions. For example, it asserts that social media activity does not influence employability, yet cites other studies showing its importance in patient acquisition. These are not contradictory findings per se, since they refer to different outcomes (employment vs. client-building). However, the manuscript presents them as opposing, weakening the argument. | The paragraph was adjusted according to the reviewer comment as:  “More shocking results were that social media activities and number of followers were not found to be significantly associated with being employed. Related studies found social media activity was useful for employed dentists to attract new patients [21], which is a different outcome (patient acquisition among already-employed dentists) rather than initial employability. That finding is supported by another study in Saudi Arabia that indicated social media was being used in personal, professional, and business aspects among currently employed dentists [22]. It seems that social media activity is useful for dentists to gain new patients after employment but not associated with becoming employed in this sample.” |
| Likewise, the phrase “more likely to be employed” is used repeatedly despite the convenience sampling and lack of controls for confounding variables. At minimum, this language should be revised to indicate associations rather than predictive relationships. | All these phrases were replaced with association as indicated above. |
| Recommendations and Limitations The authors acknowledge several limitations at the end of the paper. However, many of the inferences and policy recommendations throughout the manuscript do not reflect these limitations. If the authors are to advocate for policy changes, such as adjustments to hiring practices or salary structures, those claims should be based on more rigorous and representative data, or at least tempered in tone. | We hope now with all the adjustment made above, that the language now become very careful not to over-estimate our results, so the reader or policy maker now is more aware of the limitation of such results.  We also, highlighted this point at the end of the discussion:  “Also, conducting a national study using a probability-based random sample would inform more actionable policy decisions, as our recommendations based on our data are exploratory and should be treated as considerations for pilot testing and further study, not prescriptive policy.” |
| While the manuscript brings important attention to a growing workforce issue in Saudi Arabia, its current form requires major revision. The authors should clarify their methodology, temper their conclusions, and improve the transparency of their questionnaire design. Future revisions should also avoid overstatement of findings and better align interpretation with methodological constraints. | Thank you for the thoughtful review. We have substantially revised the manuscript to (i) clarify our methodology and recruitment (including non-probability sampling), (ii) improve transparency of questionnaire design and pilot changes, and (iii) temper conclusions by using association language and aligning interpretation with methodological constraints. We believe these revisions address the concerns and strengthen the paper. |
| **Reviewer 2** |  |
| Keywords must be based on MESH TERM. | We have changed them into:  Dentists; Employment; Health Workforce; Job Satisfaction; Saudi Arabia |
| There is no report on the validity and reliability of the questionnaire. | Thank you for that, here is the changes:  “The questionnaire was then sent in a pilot round to 17 participants (dental professionals) to validate the content, understanding, logic, syntax, flow, spelling, and grammar. Feedback led to simplify items’ wording and instructions, expand and standardize response options to avoid confusion and overlapping. Some items were removed based on the feedback on the length of the original questionnaire, to reduce response burden. Other items were reordered to improve logic and flow. This step was mandatory to ensure the face validity of the questionnaire.” |
| In the discussion section, the beginning should be different and it is not necessary to write exactly the conclusion contents. | Thank you for your comment, the first paragraph was now replaced by this:  “This study interprets employment patterns among recent Saudi dental graduates rather than restating all estimates. The overall picture is that employment was associated with time since internship completion and selected skills (notably sales), with sector differences in salary satisfaction, while several commonly presumed factors (e.g., gender, social media activity) showed no association. These observations are intended to guide hypotheses and further direction of investigations rather than to prescribe policy. In this convenience sample, all results reflect bivariate associations and should be read as preliminary. They are not causal and not population-level estimates.” |
| The discussion needs more attention and revision, and relevant articles should be used, not necessarily related to the country of Saudi Arabia. | The discussion section was revised, and many changes were conducted now. As, the suggested unemployment problem of dentists is more related to Saudi Arabia and not other countries, most of the studies compared the results with Saudi students. Nevertheless, the discussion included studies from Romania (18), South Africa (19) and USA (20,23). |
| References should be rechecked and matched to the journal format. | All reference were checked and adjusted to meet Plos One format. |
| Questionnaire should be added. | The questionnaire was added as supporting information file.  “The questionnaire of this study is available as a supporting information file in S2.” |
